# Prospective evaluation of metabolic intratumoral heterogeneity in patients with advanced gastric cancer receiving palliative chemotherapy

**DOI:** 10.1038/s41598-020-78963-2

**Published:** 2021-01-12

**Authors:** Shin Hye Yoo, Seo Young Kang, Jeesun Yoon, Tae-Yong Kim, Gi Jeong Cheon, Do-Youn Oh

**Affiliations:** 1 Department of Internal Medicine, Seoul National University Hospital, 101 Daehak-ro, Jongno-gu, Seoul, 03080 South Korea; 2 Department of Nuclear Medicine, Seoul National University Hospital, Seoul, South Korea; 3 Department of Molecular Medicine and Biopharmaceutical Science, Graduate School of Convergence Science and Technology, Seoul National University, Seoul, South Korea; 4 Cancer Research Institute, Seoul National University College of Medicine, Seoul, South Korea

**Keywords:** Cancer imaging, Gastrointestinal cancer

## Abstract

Although metabolic intratumoral heterogeneity (ITH) gives important value on treatment responses and prognoses, its association with treatment outcomes have not been reported in gastric cancer (GC). We aimed to evaluate temporal changes in metabolic ITH and the associations with treatment responses, progression-free survival (PFS), and overall survival (OS) in advanced GC patients. Eighty-five patients with unresectable, locally advanced, or metastatic GC were prospectively enrolled before the first-line palliative chemotherapy and underwent [^18^F]FDG PET at baseline (TP1) and the first response follow-up evaluation (TP2). Standardized uptake values (SUVs), volumetric parameters, and textural features were evaluated in primary gastric tumor at TP1 and TP2. Of 85 patients, 44 had partial response, 33 had stable disease, and 8 progressed. From TP1 to TP2, metabolic ITH was significantly reduced (*P* < 0.01), and the degree of the decrease was greater in responders than in non-responders (*P* < 0.01). Using multiple Cox regression analyses, a low SUV_max_ at TP2, a high kurtosis at TP2 and larger decreases in the coefficient of variance were associated with better PFS. A low SUV_max_ at TP2, larger decreases in the metabolic tumor volume and larger decreased in the energy were associated with better OS. Age older than 60 years and responders also showed better OS. An early reduction in metabolic ITH is useful to predict treatment outcomes in advanced GC patients.

## Introduction

Gastric cancer (GC) is the fifth most common cancer worldwide with high disease burden^1^, although the incidence and death rate have steadily declined^2^. GC rates are the highest in Eastern Asia, especially in the Republic of Korea, China, and Japan^1^. In the Republic of Korea, the crude mortality rate and age-standardized mortality rate for GC were shown to be 16.2 and 8.3 per 100,000, respectively in 2016^3^. Especially for advanced disease, palliative chemotherapy, and targeted therapy are essential to prolong the survival and to improve quality of life^4^.

In GC, [^18^F]fluoro-2-deoxy-d-glucose (FDG) positron emission tomography-computed tomography (PET-CT) is routine tool used to determine baseline staging, as well as CT and endoscopic ultrasound. Although [^18^F]PET-CT has higher accuracy rather than CT alone, low sensitivity to primary tumors and lymph nodes limits its role in preoperative close examination. On the other hand, the role of [^18^F]PET-CT in GC is currently regarded as beneficial for predicting treatment responses and determining prognostic information^5–8^, although it was a matter of debate^5^. Previous studies^7,9,10^ reported that standardized uptake values (SUV) or volumetric parameters, such as the metabolic tumor volume and total lesion glycolysis, are associated with treatment responses and prognoses. In addition, as intratumoral heterogeneity (ITH) has been reported to be implicated in treatment failure, higher metastasis potential, there is a growing interest in the development of new imaging strategies to assess intratumoral metabolic heterogeneity using [^18^F]FDG PET-CT. Recently several studies have reported that intratumoral FDG uptake heterogeneity, which represents metabolic ITH, is an important prognostic and predictive factor in several cancer types, such as non-small cell lung cancer, pancreatic cancer and gynecologic cancer^11–17^. However, detailed studies on metabolic ITH in GC patients are lacking. Radiomics studies examining the relationships between radiomic features and prognoses, treatment responses, and clinical characteristics in GC patients all used computed tomography (CT) imaging^13,18^. However, metabolic ITH assessed with [^18^F]FDG PET-CT may differ slightly from that measured by CT in that it evaluates the heterogeneity of tumor metabolism.

Therefore, we aimed to evaluate metabolic ITH with [^18^F]FDG PET-CT in prospectively enrolled advanced GC patients receiving palliative chemotherapy. We also sought to investigate the association of metabolic ITH with progression-free survival (PFS) and overall survival (OS).

## Results

### Patient characteristics

**Figure 1:**
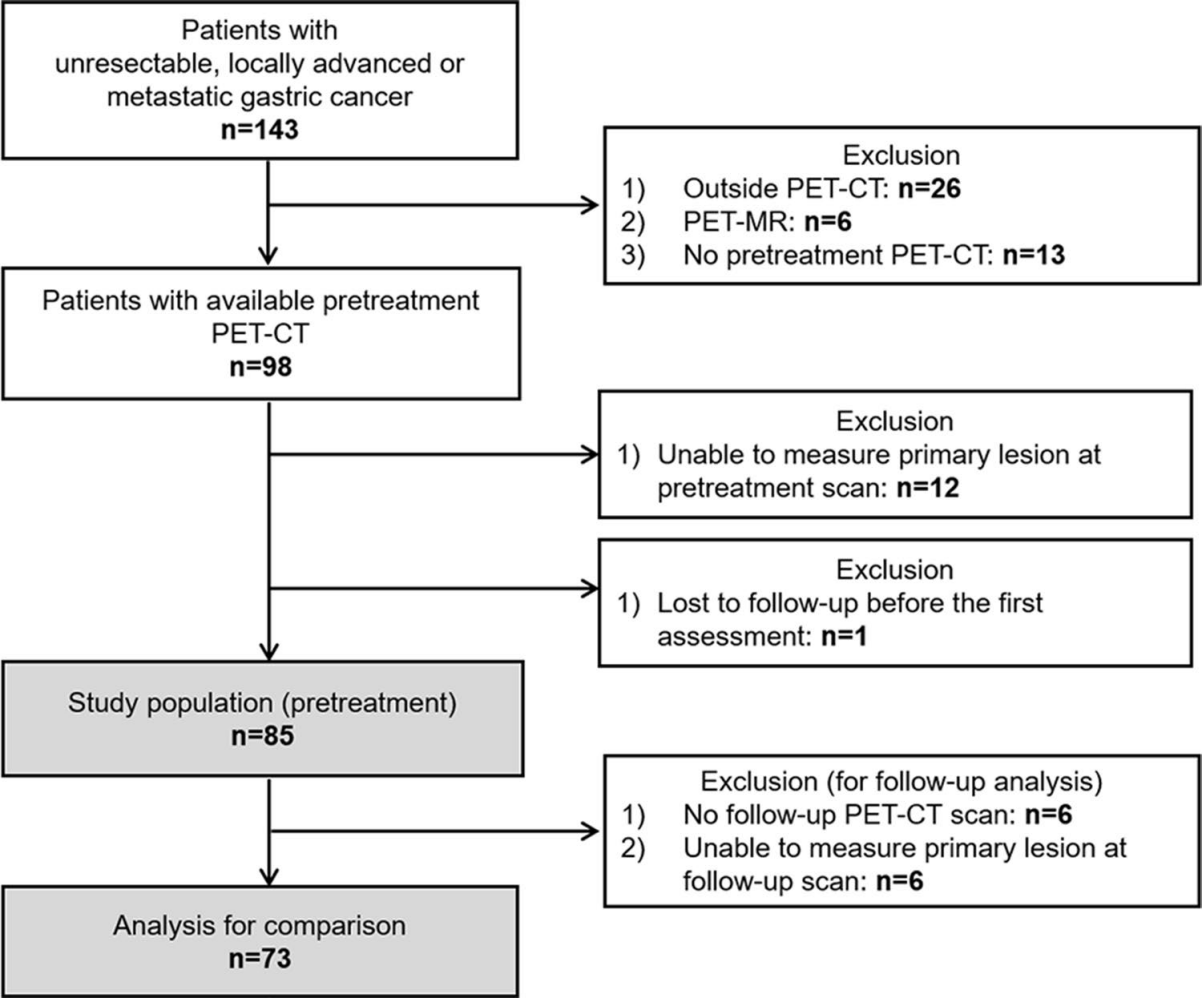
Flow chart of study participant enrollment.

Eighty-five patients were enrolled in the study consecutively from October 2013 to May 2018 (Fig. 1). Patient baseline characteristics are listed in Table 1. The median age was 59 years (range 28–81 years), and 64.7% were men. Most tumors were adenocarcinoma (81.2%); poorly cohesive carcinomas represented the rest of the tumors (18.8%). HER2 positivity was seen in 16.5% of the patients. Among 85 patients, 44 were responders with PR, and 41 were non-responders, of which 33 had stable diseases and 8 had progressive diseases. The best objective response rate was 51.8% (95% CI 40.7–62.7%), and the disease control rate was 89.5% (95% CI 83.0–96.0%). Among 44 with PR, 3 had stable diseases at TP2 and further achieved PR at later imaging follow-up. Thus, the objective response rate at TP2 was 48.2% (95% CI 37.3–59.3%). The median PFS and OS were 7.3 months (95% CI 5.4–8.2 months) and 11.5 months (95% CI 8.6–14.5 months), respectively.

**Table 1:** Baseline characteristics of the study population.

Variables	*N*	%
**Age (year), median (range)**	59	28–81
≥ 60	42	49.4
< 60	43	50.6
**Gender**		
Male	55	64.7
Female	30	35.3
**Initial disease status**		
Locally advanced, unresectable	3	3.5
Metastatic	82	96.5
**T classification**		
T2	8	9.4
T3	14	16.5
T4	63	74.1
**N classification**		
N0	11	12.9
N1	11	12.9
N2	21	24.7
N3	42	49.4
**Comorbidity**		
Hypertension	23	27.1
DM	6	7.1
**The Eastern Cooperative Oncology Group performance status**		
0	25	29.4
1–2	60	70.6
**Body-mass index (kg/m**^**2**^**), median (range)**	21.5	15–31.6
≥ 25	14	16.5
20–24.9	47	55.3
< 20	24	28.2
Carcinoembryonic antigen (ng/mL), median (range)	3.05	0.5–5420
Carbohydrate antigen 19–9 (U/mL), median (range)	23.9	1–41,600
White blood cell (× 10^3^/μL), median (range)	7320	4260–19,410
Total bilirubin (mg/dL), median (range)	0.5	0.2–1.4
Albumin (mg/dL), median (range)	3.6	2.2–4.7
**Pathology**		
Adenocarcinoma		
Poorly differentiated	36	42.4
Moderately differentiated	30	35.3
Well-differentiated	3	3.5
Poorly cohesive carcinoma (Signet ring cell carcinoma)	16	18.8
**HER2 status**		
Positive	14	16.5
Negative	71	83.5
**First-line chemotherapy**		
**Regimen**		
Capecitabine/Oxaliplatin or Folfirinic acid/Fluorouracil/Oxaliplatin	68	80.0
Capecitabine/Cisplatin/Trastuzumab	13	15.3
Other^a^	4	4.7
Duration of chemotherapy (month), median (range)	5.4	0.6–58
Number of cycles, median (range)	7	2–57
**Overall best response**		
Partial response	44	51.8
Stable disease	33	38.8
Progressive disease	8	9.4
**Best response at TP2**		
Partial response	41	48.2
Stable disease	36	42.4
Progressive disease	8	9.4
Time to best response (month), median (range)	1.44	0.9–5
Cycles till best response, median (range)	3	2–6
Time from TP1 to TP2 (day), median (range)	43	27–68
Progression-free survival (month), median (95% confidence interval)	7.3	5.4–8.2
Overall survival (month), median (95% confidence interval)	11.5	10.1–14.5
Follow-up duration (month), median (range)	11.7	1.8–57.2

^a^Other regimen includes capecitabine (n = 3) and folfirinic acid/cisplatin plus pembrolizumab (n = 1).

### Distributions of the parameters at TP1 and TP2, and parameter changes during first-line chemotherapy

From TP1 to TP2, significant changes occurred for most variables except for skewness, kurtosis, and sphericity. The values of metabolic and radiomic parameters except for energy, contrast_GLCM_, correlation, contrast_NGLDM_ and busyness decreased with chemotherapy (Supplementary Table S1).

### Comparisons of parameters between responders and non-responders

**Figure 2:**
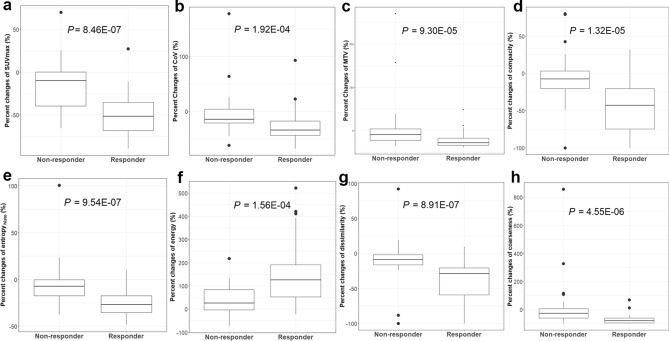
The percentage of changes in metabolic parameters that relate to best responses: SUV_max_ (**a**), CoV (**b**), MTV (**c**), compacity (**d**), entropy_Histo_ (**e**), energy (**f**), dissimilarity (**g**), and coarseness (**h**). *CoV *coefficient of variance, *Histo *histogram, *SUV *standardized uptake value, *MTV *metabolic tumor volume.

Comparisons of the metabolic and radiomic parameters between responders and non-responders were listed in Supplementary Table S2. At TP2, significant differences in most variables except skewness, kurtosis, sphericity, contrast_GLCM_, correlation, and busyness were observed between the two groups, whereas no differences were observed at TP1. The percent change between TP1 and TP2 for the same variables was also significant. The degree of decrease in SUV_max_, SUV_peak_, total lesion glycolysis, CoV, metabolic tumor volume (MTV), entropy_Histo_, compacity, entropy_GLCM_, dissimilarity, coarseness, and contrast_NGLDM_ was greater in responders than in non-responders (Fig. 2). Contrast_NGLDM_ has increased more than fivefold for non-responders, suggesting a significant increase in metabolic ITH for non-responders compared to responders.

### Progression-free survival and overall survival as predicted by pathoclinical, metabolic and radiomic parameters

**Figure 3:**
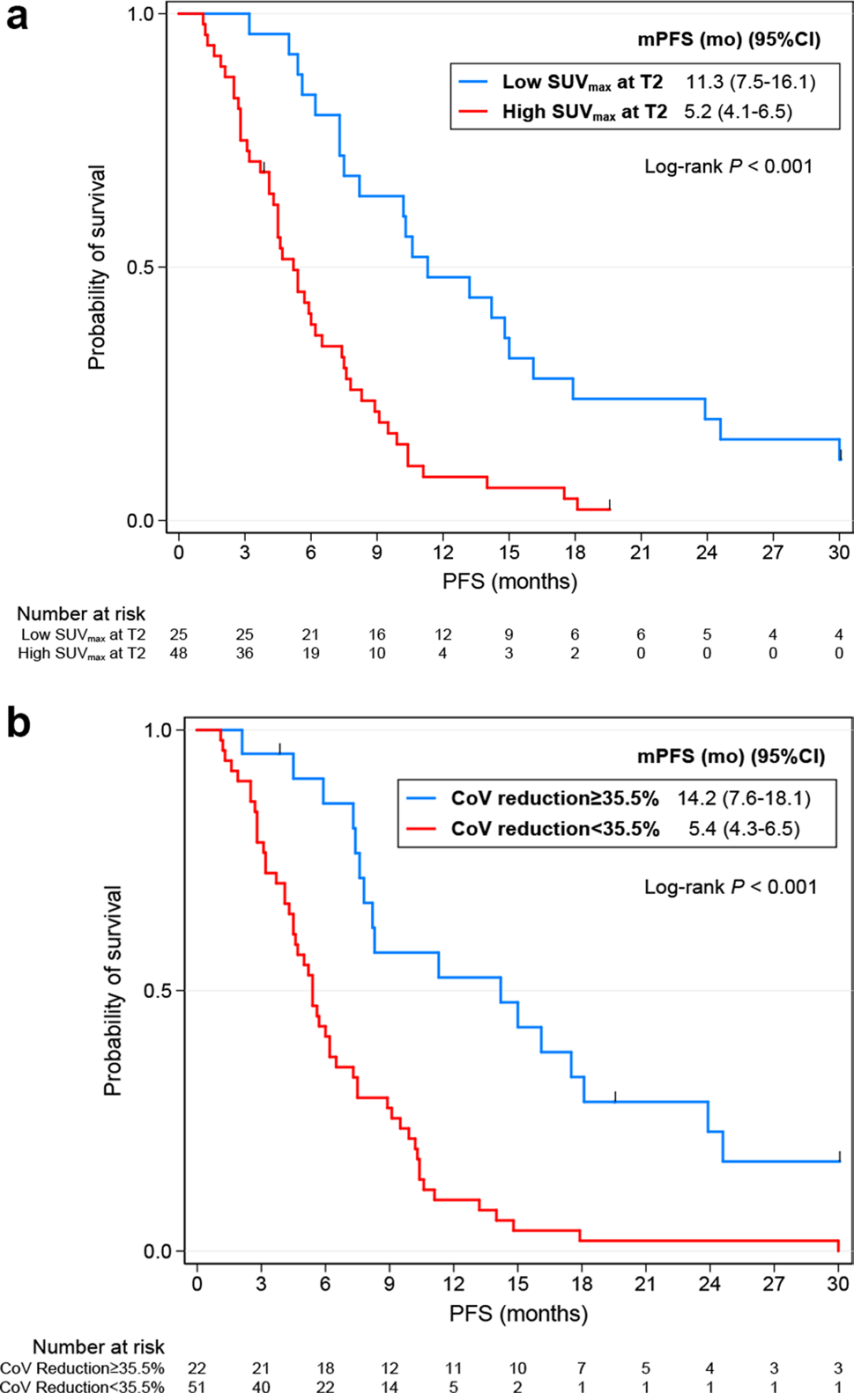
Kaplan–Meier survival curves for progression-free survival according to SUV_max_ at TP2 (**a**) and the percent change in CoV (**b**). *SUV *standardized uptake value, *CoV *coefficient of variance;

**Figure 4:**
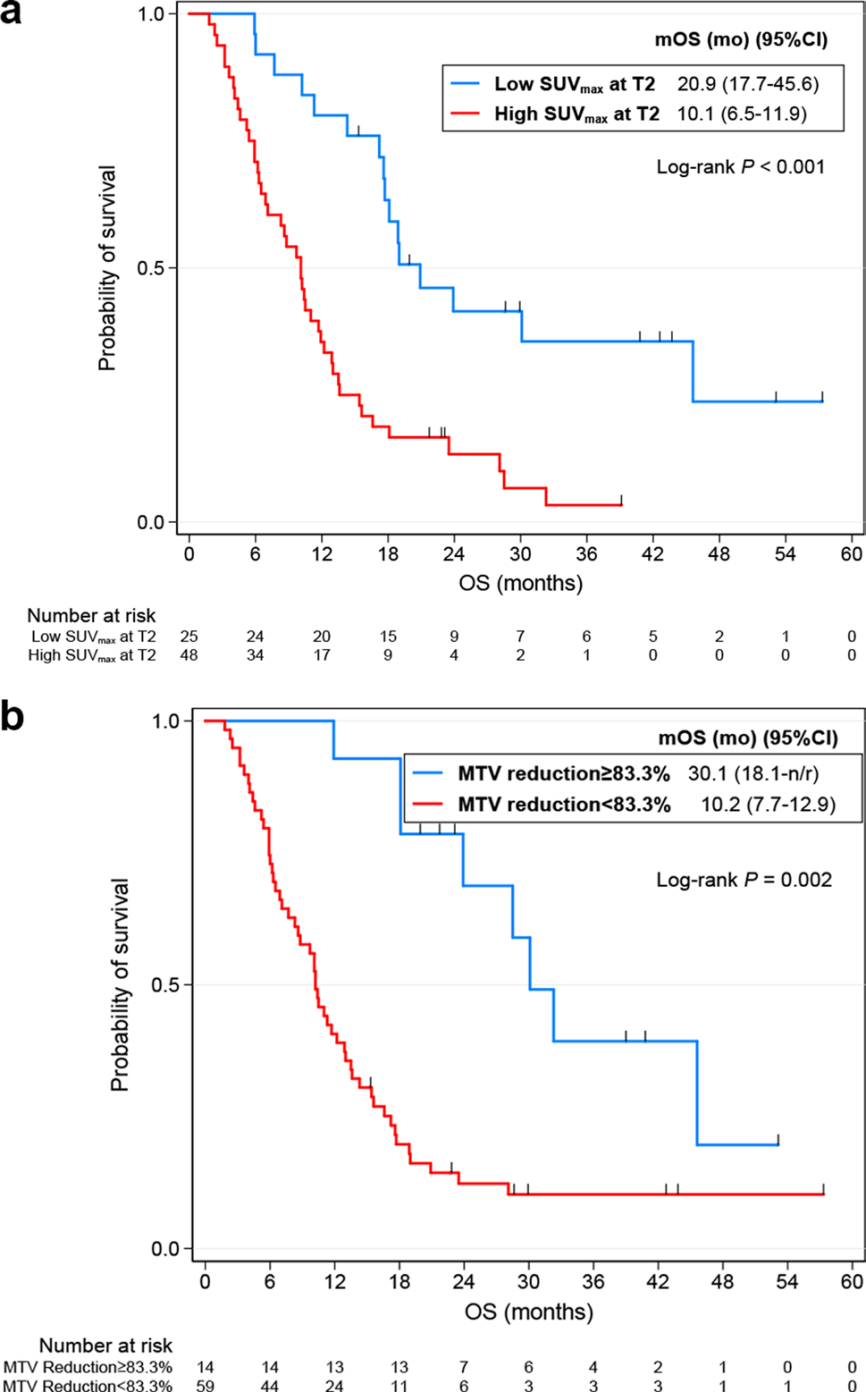
Kaplan–Meier survival curves for overall survival according to the SUV_max_ at TP2 (**a**) and percent change in MTV (**b**). *SUV *standardized uptake value, *MTV *metabolic tumor volume.

The univariate Cox regression analysis of pathoclinical variables for PFS and OS was described at Supplementary Table S3. ECOG, CEA, best response and best response at TP2 were associated with poor PFS, while pathology, ECOG, best response and best response at TP2 were correlated with poor OS. Especially, patients who achieved partial response showed prolonged PFS and OS compared to those with stable disease or progressive disease (Supplementary Fig. S2). The optimal cut-off values and corresponding AUC values for PFS and OS of metabolic and radiomic parameters were shown at Supplementary Table S4. Metabolic and radiomic parameters were divided into groups of high and low values according to their optimal cut-off values and then the continuous variables were transformed into binary variables. Supplementary Table S5 shows multiple significant metabolic and radiomic parameters at TP1, TP2 and delta by the univariate Cox regression analysis. Although only one metabolic variable, SUV_peak_, were significant at TP1 for PFS, all metabolic variables of TP2 and delta for PFS and OS were statistically significant. In addition, a number of radiomics variables were also significant at TP2 and delta for PFS and OS, compared to only a few significant ones at TP1. A correlation matrix map was used to diagnose collinearity of metabolic and radiomic parameters, and variables with r values of 0.8 or higher were excluded (Supplementary Fig. S1). Finally, the following variables remained and were used for analysis: SUV_max_, CoV, MTV, kurtosis, energy, contrast_GLCM_, correlation, entropy_GLCM_, contrast_NGLDM_, and busyness. The predictability of radiomic parameters in TP1, TP2, delta and combined variables was evaluated using C-index, and TP2 + delta model showed the best performance (Supplementary Table S6). In addition, it was confirmed that the predictive ability was improved when the pathoclinical parameters were included in this model (Supplementary Table S6; Supplementary Figs. S3 and S4 for PFS and OS, respectively). The trend of the performance of the models in 77 patients who did not progress at TP2 was also similar to those in overall patients (Supplementary Table S7). The final model for PFS by multivariable Cox regression showed that baseline CEA value (HR 2.23; 95% CI 1.19–4.16; *P* = 0.012), high SUV_max_ at TP2 (HR 2.7; 95% CI 1.30–5.60; *P* = 0.007), low kurtosis at TP2 (HR 0.42; 95% CI 0.20–0.88; *P* = 0.022), and the lesser decrease of CoV (HR 3.08; 95% CI 1.16–8.17; *P* = 0.024) were independently associated with poor PFS (Table 2; Fig. 3). For OS, age (HR 0.38; 95% CI 0.20–0.70; *P* = 0.002), best response (HR 2.57; 95% CI 1.28–5.14; *P* = 0.008), high SUV_max_ at TP2 (HR 2.59; 95% CI 1.24–5.40; *P* = 0.011), the lesser decrease of MTV (HR 3.95; 95% CI 1.45–10.74; *P* = 0.007), and the lesser decrease of energy (HR 3.62; 95% CI 1.10–11.88; *P* = 0.003) were associated with poor survival (Table 2; Fig. 4).The performance of each models was compared in the Supplementary Table S6.

**Table 2:** Pathoclinical and radiomics variables selected by Lasso Cox regression model and identified by multivariable Cox regression model.

Variables	PFS					OS				
	Lasso regression		Multivariable Cox regression			Lasso regression		Multivariable Cox regression		
	Coefficient	SE	HR	95% CI	P*	Coefficient	SE	HR	95% CI	P*
**Pathoclinical**										
Age	− 0.24	0.296	0.58	0.32–1.06	0.076	− 0.516	0.416	0.38	0.20–0.70	**0.002**
ECOG	0.253	0.315	1.5	0.81–2.75	0.194	0.377	0.44	1.8	0.94–3.48	0.078
CEA	0.463	0.348	2.23	1.19–4.16	**0.012**	0.141	0.348	1.34	0.71–2.51	0.366
Best response						0.41	0.385	2.57	1.28–5.14	**0.008**
**TP2**										
SUV_max_	0.697	0.428	2.7	1.30–5.60	**0.007**	0.879	0.559	2.59	1.24–5.40	**0.011**
CoV	0.066	0.444	1.48	0.55–3.96	0.440					
Kurtosis	− 0.359	0.502	0.42	0.20–0.88	**0.022**					
**Delta**										
CoV	0.881	0.513	3.08	1.16–8.17	**0.024**	0.82	0.605	1.87	0.89–3.91	0.096
MTV	0.468	0.6	2.18	0.82–5.79	0.118	0.837	0.718	3.95	1.45–10.74	**0.007**
Energy	0.0684	0.495	1.64	0.58–4.67	0.351	0.679	0.617	3.62	1.10–11.88	**0.034**
Contrast_GLCM_	0.231	0.335	1.42	0.55–3.66	0.472					
Correlation	0.345	0.447	1.53	0.56–4.15	0.399	0.157	0.562	1.02	0.41–2.55	0.959

*PFS *progression-free survival, *OS *overall survival, *SE *standard error, *HR *hazard ratio, *CI *confidence interval, *SUV *standardized uptake values, *CoV *coefficient of variation, *ECOG* The Eastern Cooperative Oncology Group, *CEA *carcinoembryonic antigen, *CoV *coefficient of variation, *MTV *metabolic tumor volume, *GLCM *gray-level co-occurrence matrix.

Bold values denote statistical significance at the *P* < 0.05 level.

*P value was generated by multivariable Cox proportional hazards regression analysis.

## Discussion

In this prospective study, we found that metabolic parameters decreased during palliative chemotherapy more in responders than in non-responders. Post-chemotherapy metabolism and metabolic changes were significantly associated with PFS and OS in patients receiving palliative chemotherapy. To our knowledge, this is the first study to show the associations of delta-radiomics assessed with [^18^F]FDG PET-CT with survival in advanced GC patients.

We found that most metabolic and radiomic parameters, except skewness, kurtosis, and sphericity had a declining trend of metabolism and heterogeneity throughout chemotherapy, which is consistent with the results of other studies in advanced pancreatic cancer^19^ and non-small cell lung cancer^11,12^. This negative trend over time implies that dominant tumor subclones might disappear during treatment, and the heterogeneity of overall tumor clones could be decreased^20^.

**Figure 5:**
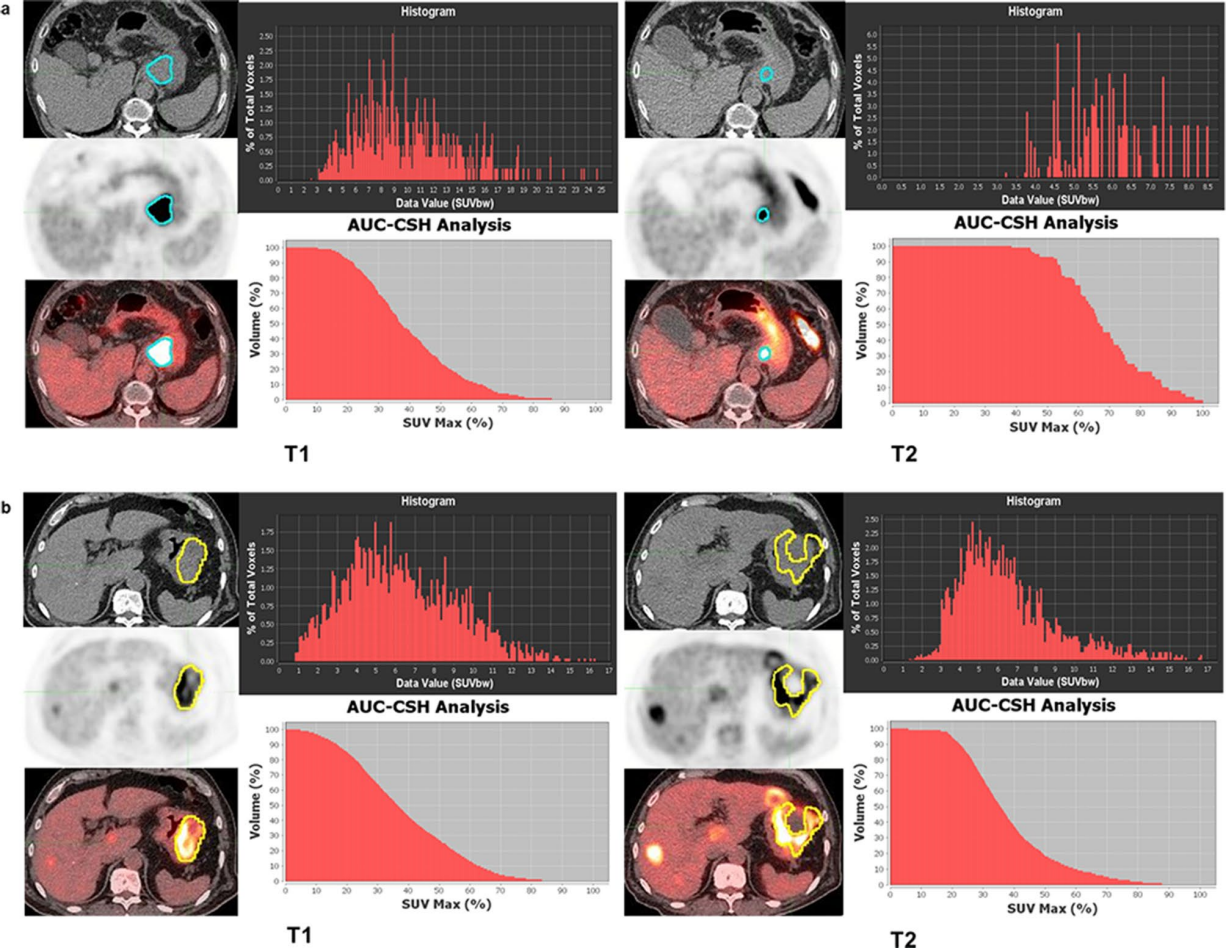
Representative cases showing clinical significance of metabolic ITH changes from [^18^F]FDG-PET-CT. Case (**a**) is a 74-year-old male patient with PFS of 19.5 months and OS of 19.8 months. The histogram and AUC-CSH showed decreased metabolic ITH after palliative chemotherapy. Case (**b**) is a 72-year-old male patient with PFS of 1.1 months and OS of 2.3 months, suggesting very poor prognosis. The histogram and AUC-CSH represent no significant change of metabolic ITH after palliative chemotherapy. *AUC-CSH *area under the curve of the cumulative standardized uptake value volume histogram, *[*^*18*^*F]FDG-PET-CT *[^18^F]fluoro-2-deoxy-d-glucose positron emission tomography-computed tomography, *ITH *intratumoral heterogeneity, *OS *overall survival, *PFS *progression free survival, *ROI *region of interest.

In our study, the levels of heterogeneity at TP2, and the percent changes in heterogeneity, but not pretreatment heterogeneity, were lower in responders than in non-responders. The associations of metabolic heterogeneity assessed using PET-CT with responses to chemotherapy have not been well-studied in GC patients, although a few studies have reported that an early metabolic response, using metabolic parameters, is associated with positive treatment outcomes^21^ or that responses measured with PET-CT could be helpful for deciding on treatment strategies^6^. Giganti et al*.* revealed that pretreatment radiomic features such as entropy, ranges, and root mean squares predicted the responses to neoadjuvant chemotherapy in GC patients. However, our findings showed an association of radiomic features measured at the early assessment time (TP2) or the delta-radiomic features with treatment responses, which suggest that responses could be associated with the heterogeneity parameter trends, rather than parameters themselves at pretreatment. This association of a change in tumor composition with treatment responses could provide useful information, in addition to the early metabolic responses assessed by SUV values^21^. Figure 5 depicts representative cases showing clinical significance of reduction in metabolic ITH. Both cases showed similar levels of metabolic ITH before treatment, but metabolic ITH of a patient with good post-treatment outcomes manifested significant decrease, while metabolic ITH of patients with very poor outcomes has not changes much. Similarly, the cases of Supplementary Fig. S5 also illustrate that changes in the radiomic features are related to the outcomes of the patients.

To date, the prognostic impact of texture parameters has primarily been investigated in patients with surgically resected GCs, using a CT texture analysis. To our knowledge, only one study, including 26 patients diagnosed as HER2-positive advanced GC and treated with trastuzumab, focused on advanced GC patients^22^. In that study, patients with higher contrast, correlation, and variance at pretreatment had longer OS than those with lower contrast, correlation, and variance. These results were consistent with our findings that pretreatment contrast_GLCM_ and entropy was associated with OS. However, these values were excluded from the final model because they showed a relatively low C-index compared to other texture parameters of TP2 and delta.

In our study, delta values of CoV were an independent factor for PFS, and MTV and energy were for OS. These findings emphasize the importance of delta-radiomics features, especially those that represent ITH changes, in addition to MTV and CoV which has already been reported for its importance. The predictive role of delta-radiomics features on PFS has been consistently observed in studies of other cancer types^13,19,23^, although the association of pretreatment texture features with PFS was only addressed in studies of GC patients^24–26^. The biologic mechanisms connecting ITH changes and tumor progression have not been elucidated to date. Greater reductions in metabolic ITH indicate that the tumor and surrounding microenvironment become more unified and that variations in grey-level distributions become minimized. Previous reports^27,28^ suggest that ITH could be associated with cell cycling pathways and immune infiltrate distributions so that changes in ITH could reflect changes in the tumor microenvironment.

The National Comprehensive Cancer Network guideline^29^ suggests that [^18^F]FDG PET-CT can be performed as clinically indicated in diagnosis, response assessment, and follow-up and surveillance in GC. Although it is premature to change treatment options based solely on the results from our models, this study can suggest an additional role of [^18^F]FDG PET-CT, which can be used for risk stratification of patients, by presenting an evaluation method of ITH in GC and proving an important role in prognostic evaluation. Risk stratification of progression is crucial before confirming the definitive progression by clinical and radiologic criteria because the next treatment strategy should be planned in advance. In this context, the potential clinical utility of the model could be suggested. When assessing at TP2 and constructing the TP2+ delta model, it is possible to predict those who are most likely to ultimately show short PFS in 1st-line palliative chemotherapy. Especially, regardless of the patients who progress at TP2, the model is able to predict high-risk group for progression. Although we did not calculate radiomics score, several studies using radiomics score and performing risk stratification by the score^24,25^ showed that the patient in high-risk group could be identified. In such a case, assessing the clinical symptoms/signs cautiously by close follow-up can be beneficial, and the next treatment plan could be prepared. Lack of evidence showing the cost-effectiveness of these models limits the application of our results to general treatment schemes. Although our study did not provide the cost-effectiveness analysis, the cost-effectiveness of model using radiomics variables needs to be studied to ensure it to provide good value for cost compared with the other currently available biomarkers after biological and clinical validation.

This study has several limitations. First, we only assessed the tumor metabolism of primary gastric lesions, so spatial metabolic ITH between primary and metastatic lesions was not considered. Next, the patients had received different therapies, which might have caused confounding effects. However, an insignificant association of the treatment variable in the univariate analysis was observed. Third, SUV and ITH parameters are highly dependent on the instrumentation and reconstruction parameters used during the study. Extrapolation of the discrete values of data in the study to other scanners and reconstruction would require care. Fourth, motion of the tumors (gastric peristalsis) understandably cannot be accounted for in this data, and may have some influence on measures of heterogeneity. Fifth, we only included a training set without a validation cohort. Further external validations are needed.

Despite these limitations, we built a multivariable regression model using pathoclinical and radiomic parameters. It has been reported that models with both parameters had better predictive accuracy in patients with surgically resected GC than those with either parameter assessed alone^24,26^. Similarly, the multivariable model used in our study could predict PFS and OS in advanced GC patients. Our study also highlighted the predictive role of delta-radiomics, whereas previous studies only assessed pretreatment metabolic parameters^22^, which suggest that clinicians should consider metabolic ITH changes as a predictive biomarker when deciding on GC patient therapies.

In conclusion, our study demonstrated that metabolic ITH levels decreased during palliative chemotherapy, and early decreases in metabolic ITH can predict positive responses to palliative chemotherapy, PFS, and OS in advanced GC patients.

## Methods

### Patient enrollment and data measurements

We prospectively enrolled patients who were diagnosed with advanced GC (unresectable locally advanced or metastatic disease) and planned for first-line palliative chemotherapy. All patients underwent [^18^F]FDG PET-CT and contrast-enhanced CT scanning before initiating chemotherapy (TP1) and at the time of the first tumor response evaluation (TP2), usually after 2 or 3 cycles of chemotherapy, and then, serially for each subsequent response evaluation, when possible. The study protocol was reviewed and approved by Seoul National University Hospital Institutional Review Board (H-1307-132-508). We conducted the study in accordance with the principles of the Declaration of Helsinki. Informed consent was obtained from all individual participants included in the study. Informed consent to publish identifying information and images for Fig. 5 was also obtained from the corresponding participants. Cases with primary gastric lesions that could not be examined were excluded. Case lost to follow-up before TP2 was excluded (Fig. 1). Pathoclinical characteristics, chemotherapeutic responses assessed using the Response Evaluation Criteria in Solid Tumors version 1.1^30^, and survival data were collected. Human epidermal growth factor receptor 2 (HER2) positivity was defined as HER2 immunohistochemistry 3+ or immunohistochemistry 2+ and FISH-positive (HER2/CEP17 (centromere enumerator probe 17) ratio ≥ 2) using the PathVysion HER-2 DNA probe kit (Vysis).

### The [^18^F]FDG PET-CT protocol

All patients fasted for at least 6 h prior to the intravenous administration of [^18^F]FDG (5.18 MBq/kg), resulting in a serum glucose concentration of 150 mg/dL. [^18^F]FDG PET-CT was performed at 1 h after the injection using a dedicated scanner (Biograph 40 TruePoint; Siemens). After a low-dose CT scan for attenuation correction (120 kVp; tube current, 60–210 mA; beam collimation, 40 mm (0.625 × 94); pitch factor, 0.516:1; coverage speed, 41.24 mm/s; and tube rotation time, 0.5 s), a consecutive emission scan was acquired in 3 dimensions (5–6 bed positions, 2.5 min/bed position, 5 mm slice thickness 21.6-cm increments). PET images were reconstructed on to a matrix of 128 128 with voxel size 4 × 4 × 4 mm^3^ using 3-dimensional ordered-subset expectation–maximization (2 iterations, 21 subsets). For post-processing, a 5.0-mm Gaussian filter was used to reduce noise and smoothen image quality.

### Tumor segmentation and texture analyses

Our study followed and adhered to the Image Biomarker Standardization Initiative (IBSI) guidelines^31^, and the software LifeX (version 4.0) compliant with IBSI was used for analysis^32^. Tumor delineation, and metabolic parameter and texture analyses were performed by a nuclear medicine specialist without knowledge of the clinical information. The volume of interest of the primary gastric lesion was automatically defined using PET Edge, a gradient-based delineation tool in MIM Encore (version 4.1; MIM Software Inc.; Cleveland, OH, USA) used for tumor segmentation. SUV parameters and volumetric parameters, including the metabolic tumor volume and total lesion glycolysis, were extracted from the volume of interest. The coefficient of variance (CoV) is defined as the standard deviation of the SUVs divided by the mean SUV (SUV_mean_) and correlates positively with the degree of heterogeneity in the volume of interest^14^. For texture analysis, we imported [^18^F]FDG PET-CT images and delineation data to LifeX. Each [^18^F]FDG PET-CT image was resampled into a 64-level grayscale by a fixed-bin-width method with 0.3-SUV-unit scaling, from the minimum to maximum SUV values of 0–20. We included the histogram and shape indices as first-order parameters, gray-level co-occurrence matrix (GLCM) indices as second-order parameters, and the neighboring gray-level dependence matrix (NGLDM) indices as higher-order parameters^13^ (Supplementary Table S8). For quality assurance a Radiomics Quality Score was calculated^33^ for this study. The Radiomics Quality Score of our study was 13, which was 36% of the ideal score of 36. This was slightly higher than the average score of 11 assessed in the systemic review^34^.

### Statistical analysis and modeling

The paired *t*-test for parametric analyses and the Wilcoxon signed-rank test for non-parametric analyses were performed to analyze the difference between the TP1 and TP2 parameters of SUV and texture analyses. The Student *t*-test or Mann–Whitney test was used to compare variables between responders (complete response or partial response) and non-responders (stable disease or progressive disease). To reduce the risk of false discovery and correct random events, false discovery rate was calculated using Benjamini Hochberg procedure, a statistical approach for multiple comparisons.

OS was defined as the time from initiating first-line chemotherapy until death, and the PFS was defined as the time from the initiating first-line chemotherapy to disease progression or any cause of death. Overall response was defined as complete or partial responses as their best overall response based on the RECIST. All variables were tested using the univariate Cox proportional-hazards model. The optimal cut-off of the SUV and texture analysis parameters, discriminating a high or low result in terms of PFS and OS, was determined using time-dependent receiver-operating characteristic (ROC) curve analyses. It is more appropriate to apply the cumulative sensitivity/dynamic specificity definitions when there is a specific time of interest that is used to discriminate between individuals experiencing the event and those event-free prior to the specific time. Cumulative sensitivity/dynamic specificity definition has commonly been used by clinical applications. We used 1-year span as a specific time horizon in the time-dependent ROC analysis.

Prior to performing multivariate logistic regression, collinearity diagnostics were performed using a correlation matrix map. In the end, only 30 parameters were included in the further analysis, excluding those with an r value of 0.8 or higher. Considering high dimension of variables and model overfitting, the least absolute shrinkage and selection operator (LASSO) Cox regression analysis under the bootstrap conditions was performed to evaluate the discrimination performances of different models using texture parameters. Five hundred iterations were performed with random data resampling between runs. When comparing predictive models using the texture parameters from TP1, TP2, and delta, the model containing the variables from TP2+ delta showed the best performance. Therefore, the variables from TP2+ delta were selected for the final regression models. To evaluate the predictive ability of combined models using pathoclinical, radiomics, pathoclinical + radiomics features, Harrel’s concordance index (C-index) were calculated and compared. Finally, the predictive values from pathoclinical and texture parameters were further validated using LASSO multiple regression analysis associated with PFS and OS. For subgroup analysis, in order to find out whether the models would predict well when the patients with progressive disease at TP2 were removed, the performance of the model of patients who did not progress at TP2 was revealed in a same manner of overall patients.

Analyses were performed using STATA, version 15 (StataCorp LP), and R software (version 3.6.0, www.Rproject.org). The area under the curve (AUC) was calculated by the “survivalROC” package in R. LASSO was conducted using the “glmnet” package, and the “boot” package was used for bootstrapping. All statistical tests were two-sided, with a significance level of 0.05.

A TRIPOD Checklist in accordance with strict multivariate model building and reporting guidelines (https://www.equator-network.org/reporting-guidelines/tripod-statement/) has also been provided in Supplementary Table S9, further verifying the integrity of the work^35^.

## Supplementary Information

Supplementary Information.

Supplementary Figure S1.

Supplementary Figure S2.

Supplementary Figure S3.

Supplementary Figure S4.

Supplementary Figure S5.

## Data Availability

All data generated or analysed during this study are included in this published article and its Supplementary Information files.
