# Supplementary material for: Prospective evaluation of metabolic intratumoral heterogeneity in patients with advanced gastric cancer receiving palliative chemotherapy
Source: Sci Rep. 2021 Jan 12;11:296. doi: 10.1038/s41598-020-78963-2 (PMC7804009; doi:10.1038/s41598-020-78963-2)
Supplement: Supplementary file 1 — Supplementary Information. [file 41598_2020_78963_MOESM1_ESM.docx]

**Prospective Evaluation of Metabolic Intratumoral Heterogeneity in Patients with Advanced Gastric Cancer Receiving Palliative Chemotherapy**

Shin Hye Yoo, MD, PhD^1†^, Seo Young Kang, MD, PhD^2,3†^, Jeesun Yoon, MD^1^, Tae-Yong Kim, MD^1,4^, Gi Jeong Cheon, MD, PhD^2,3^, Do-Youn Oh, MD, PhD^1,4*^

**Supplementary Figure Legends**

**Supplementary Figure S1**. Correlation matrix of radiomics variables: TP1 (a), TP2 (b), and delta (c). A representation of the correlation matrix in which coefficients are replaced by symbols according to the strength of the dependence. Positive correlations are displayed in a blue scale while negative correlations are displayed in a red scale.

**Supplementary Figure S2**. Kaplan-Meier survival curves for progression-free survival (a) and overall survival (b) according to the overall best response by the RECIST version 1.1. PFS = progression-free survival; PR = partial response; SD = stable disease; PD = progressive disease; OS = overall survival;

**Supplementary Figure S3**. Radiomics feature selection using least absolute shrinkage and selection operator (LASSO) Cox regression model for progression-free survival. LASSO coefficient profiles of the candidate radiomic features (a) were plotted. Optimal λ was identified used 10-fold cross-validation and the minimum criterion. A partial likelihood deviance from the LASSO Cox regression procedure was plotted against log(λ) (b).

**Supplementary Figure S4**. Radiomics feature selection using least absolute shrinkage and selection operator (LASSO) Cox regression model for overall survival. LASSO coefficient profiles of the candidate radiomic features (a) were plotted. Optimal λ was identified used 10-fold cross-validation and the minimum criterion. A partial likelihood deviance from the LASSO Cox regression procedure was plotted against log(λ) (b).

**Supplementary Figure S5**. Representative examples of the importance of changes in the radiological features. The first case is a 67-year-old man (a). After chemotherapy, delta changes in energy, contrast_GLCM_, and correlation increased 86.9%, 42.7%, and 254%, respectively, despite 35% drop in SUV_max_ of tumor. The patient's progression-free survival (PFS) was 5.4 months and overall survival (OS) was 5 months, showing poor prognosis. The second case is a 46-year-old man (b). Despite an 49% decrease in tumor SUV_max_ after chemotherapy, the delta changes in energy, contrast_GLCM_, and correlation increased by 231%, 51% and 564%, respectively. The patient's PFS was 8.3 months and OS was 8.3 months, showing poor prognosis. The third case is also a 46-year-old man (c). Despite an 18% increase in tumor SUV_max_ after chemotherapy, the delta changes in skewness, energy, contrast_GLCM_, and correlation decreased by 43%, 37%, 16%, and 52%, respectively. The patient's PFS was 10.4 months and OS was 13 months, showing relatively better prognosis. The last case is a 55-year-old man (d). After chemotherapy, most of the radiomic features such as skewness (85%), kurtosis (55%), entropy (3%), energy (3%), contrast_GLCM_ (23%), and entropy_GLCM_ (44%) showed a tendency to decrease. The patient's PFS was 17.5 months and OS was 32.3 months, showing good prognosis.

**Supplementary** **Table S1**. Metabolic parameters at baseline (TP1) and the first assessment (TP2), and percent changes seen

| **Parameter** | **TP1 (baseline) (*N*=85)** | | | | **TP2 (first assessment) (*N*=73)** | | | | **Percent change* (*N*=73)** | | | |  |
| --- | --- | --- | --- | --- | --- | --- | --- | --- | --- | --- | --- | --- | --- |
|  | Mean | Sd | Median | Range | Mean | Sd | Median | Range | Mean | Sd | Median | Range | *P*^†^ |
| **Conventional Indices** |  |  |  |  |  |  |  |  |  |  |  |  |  |
| SUV_max_ | 14.29 | 8.5 | 12.75 | 2.99―49.11 | 8.51 | 4.78 | 7.38 | 3.61―30.47 | -33.22 | 31.43 | -38.34 | -89.22―69.68 | **1.35E-09** |
| SUV_peak_ | 11.46 | 6.89 | 10.29 | 0―34.94 | 6.41 | 4.44 | 6.01 | 0―23.85 | -40.04 | 34.36 | -41.3 | -100―33.34 | **2.36E-13** |
| TLG | 397.64 | 498.57 | 210.39 | 5.87―2138.52 | 169.7 | 334.42 | 49.9 | 3.85―2151.92 | -36.64 | 127.35 | -65.98 | -99.47―879.96 | **5.19E-08** |
| CoV | 0.27 | 0.08 | 0.26 | 0.08―0.49 | 0.22 | 0.07 | 0.2 | 0.09―0.46 | -16.95 | 35.65 | -21.51 | -67.68―177.39 | **8.96E-07** |
| MTV | 49.59 | 58.93 | 30.03 | 1.21―328.89 | 30.09 | 52.66 | 10.82 | 1.12―330.39 | -26.33 | 108.24 | -56.08 | -98.31―675.18 | **1.59E-06** |
| **Histogram Indices** |  |  |  |  |  |  |  |  |  |  |  |  |  |
| Skewness | 0.53 | 0.43 | 0.45 | -0.47―2.22 | 0.42 | 0.5 | 0.33 | -0.33―1.95 | 1072.98 | 9247.5 | -43.25 | -1017.83―78965.23 | 0.052 |
| Kurtosis | 3.17 | 1.45 | 2.79 | 1.89―11.40 | 3.08 | 1.11 | 2.73 | 1.90―8.71 | 4.66 | 36.76 | 2.14 | -68.68―157.82 | 0.553 |
| Entropy _(log2)_ | 4.24 | 0.85 | 4.4 | 1.82―5.49 | 3.51 | 0.75 | 3.45 | 1.84―5.46 | -16.45 | 21.03 | -17.91 | -48.52―100.50 | **3.50E-09** |
| Energy | 0.08 | 0.05 | 0.06 | 0.02―0.33 | 0.11 | 0.06 | 0.1 | 0.02―0.30 | 96.47 | 118.76 | 67.03 | -72.53―521.01 | **1.35E-09** |
| **Shape Indices** |  |  |  |  |  |  |  |  |  |  |  |  |  |
| Sphericity | 0.92 | 0.19 | 0.95 | 0―1.08 | 0.78 | 0.37 | 0.95 | 0―1.11 | -12.57 | 36.95 | 0.06 | -100―51.71 | 0.362 |
| Compacity | 1.84 | 0.76 | 1.73 | 0―3.96 | 1.33 | 0.91 | 1.21 | 0―4.40 | -28.24 | 40.2 | -20.6 | -100―79.92 | **2.46E-07** |
| **GLCM** |  |  |  |  |  |  |  |  |  |  |  |  |  |
| Contrast | 0.32 | 0.11 | 0.31 | 0―0.64 | 0.32 | 0.17 | 0.36 | 0―0.57 | 7.99 | 55.65 | 12.27 | -100―136.09 | **0.039** |
| Correlation | 0.012 | 0.017 | 0.006 | 0―0.106 | 0.022 | 0.07 | 0.011 | 0―0.60 | 190.96 | 361.19 | 74.15 | -100―2186.76 | **8.23E-06** |
| Entropy _(log2)_ | 0.48 | 0.17 | 0.51 | 0―0.78 | 0.38 | 0.31 | 0.41 | 0―2.19 | -20.78 | 56.16 | -15.86 | -100―222.40 | **1.18E-04** |
| Dissimilarity | 2.23 | 0.6 | 2.29 | 0―3.12 | 1.61 | 0.84 | 1.86 | 0―2.98 | -27.07 | 35.99 | -17.82 | -100―91.85 | **1.35E-09** |
| **NGLDM** |  |  |  |  |  |  |  |  |  |  |  |  |  |
| Coarseness | 796.73 | 899.62 | 518.19 | 0―4655.99 | 447.14 | 804.02 | 169.81 | 0―5072.47 | -30.35 | 126.45 | -61.6 | -100―856.79 | **1.59E-06** |
| Contrast | 0.89 | 0.18 | 0.93 | 0―0.97 | 2.84 | 17.92 | 0.9 | 0―153.82 | 247.52 | 2202.02 | -2.68 | -100―18405.41 | **6.42E-04** |
| Busyness | 0.02 | 0.01 | 0.01 | 0―0.06 | 0.03 | 0.1 | 0.02 | 0―0.82 | 343.97 | 1470.79 | 56.99 | -100―825.26 | **5.96E-05** |

Sd = standard deviation; SUV = standardized uptake values; TLG = total lesion glycolysis; CoV = coefficient of variation; MTV = metabolic tumor volume; GLCM = gray-level co-occurrence matrix; NGLDM = neighboring gray-level dependence matrix;

Bold values denote statistical significance at the *P* < 0.05 level.

^*^The percent change was calculated by 100 × [value at the first assessment (TP2) minus the baseline value (TP1)] / value at the baseline (TP1)

†*P* value was generated by paired *t*-test for parametric samples or Wilcoxon signed-rank test for non-parametric samples, followed by correction for multiple comparisons by Benjamini-Hochberg method.

**Supplementary** **Table S2**. Comparison of parameters between responders and non-responders

| **Parameter** | **TP1 (baseline) (*N*=85)** | |  | **TP2 (first assessment) (*N*=73)** | |  | **Percent change* (*N*=73)** | |  |
| --- | --- | --- | --- | --- | --- | --- | --- | --- | --- |
|  | Non-responder | Responder |  | Non-responder | Responder |  | Non-responder | Responder |  |
|  | *N*=41 (48.2%) | *N*=44 (51.8%) |  | *N*=34 (46.6%) | *N*=39 (53.4%) |  | *N*=34 (46.6%) | *N*=39 (53.4%) |  |
|  | Mean (Sd) | | *P*^†^ | Mean (Sd) | | *P*^†^ | Mean (Sd) | | *P*† |
| **Conventional Indices** |  |  |  |  |  |  |  |  |  |
| SUV_max_ | 12.70 (7.19) | 15.77 (9.41) | 0.802 | 10.26 (5.73) | 6.98 (3.09) | **0.003** | -14.16 (28.96) | -49.84 (23.14) | **8.46E-07** |
| SUV_peak_ | 10.35 (5.76) | 12.50 (7.72) | 0.802 | 8.49 (4.75) | 4.59 (3.25) | **5.05E-04** | -17.61 (24.89) | -60.04 (28.97) | **1.76E-07** |
| TLG | 397.18 (515.8) | 398.07 (487.96) | 0.922 | 297.1 (446.62) | 58.63 (107.27) | **3.17E-05** | 7.84 (176.13) | -75.42 (21.78) | **8.46E-07** |
| CoV | 0.26 (0.08) | 0.28 (0.08) | 0.802 | 0.24 (0.08) | 0.19 (0.07) | **0.004** | -4.79 (39.63) | -27.54 (28.22) | **1.92E-04** |
| MTV | 54.92 (68.6) | 44.62 (48.52) | 0.913 | 50.29 (69.08) | 12.49 (20.64) | **6.71E-05** | 10.1 (144.61) | -58.09 (42.62) | **9.30E-05** |
| **Histogram Indices** |  |  |  |  |  |  |  |  |  |
| Skewness | 0.51 (0.40) | 0.55 (0.47) | 0.997 | 0.46 (0.50) | 0.39 (0.49) | 0.582 | 14.98 (385.37) | 1995.33 (12650.18) | 0.494 |
| Kurtosis | 3.19 (1.46) | 3.15 (1.46) | 0.877 | 3.13 (0.90) | 3.04 (1.29) | 0.167 | 5.48 (31.84) | 3.94 (40.98) | 0.589 |
| Entropy_(log2)_ | 4.14 (0.92) | 4.34 (0.79) | 0.802 | 3.84 (0.78) | 3.21 (0.59) | **5.32E-04** | -5.31 (23.05) | -26.16 (12.98) | **9.54E-07** |
| Energy | 0.08 (0.06) | 0.07 (0.05) | 0.913 | 0.09 (0.05) | 0.13 (0.05) | **3.54E-03** | 41.17 (61.51) | 144.69 (135.34) | **1.56E-04** |
| **Shape Indices** |  |  |  |  |  |  |  |  |  |
| Sphericity | 0.95 (0.07) | 0.89 (0.26) | 0.913 | 0.91 (0.17) | 0.68 (0.46) | 0.79 | -3.45 (17.62) | -21.19 (47.35) | 0.86 |
| Compacity | 1.93 (0.74) | 1.75 (0.78) | 0.802 | 1.82 (0.87) | 0.90 (0.71) | **6.71E-05** | -7.42 (33.96) | -47.90 (35.77) | **1.32E-05** |
| **GLCM** |  |  |  |  |  |  |  |  |  |
| Contrast | 0.34 (0.11) | 0.29 (0.11) | 0.802 | 0.36 (0.10) | 0.28 (0.20) | 0.476 | 10.28 (31.85) | 5.90 (71.27) | 0.589 |
| Correlation | 0.013 (0.018) | 0.011 (0.016) | 0.802 | 0.030 (0.10) | 0.014 (0.013) | 0.996 | 137.58 (378.89) | 241.38 (341.24) | 0.332 |
| Entropy_(log2)_ | 0.49 (0.14) | 0.48 (0.20) | 0.913 | 0.52 (0.34) | 0.25 (0.21) | **1.03E-04** | 8.85 (57.47) | -48.78 (38.02) | **4.20E-06** |
| Dissimilarity | 2.26 (0.47) | 2.21 (0.71) | 0.913 | 2.00 (0.59) | 1.27 (0.88) | **8.39E-05** | -10.00 (29.08) | -43.18 (34.73) | **8.91E-07** |
| **NGLDM** |  |  |  |  |  |  |  |  |  |
| Coarseness | 842.26 (983.24) | 754.30 (823.33) | 0.922 | 761.15 (1055.21) | 173.39 (304.56) | **6.71E-05** | 13.19 (168.45) | -71.47 (34.48) | **4.55E-06** |
| Contrast | 0.92 (0.04) | 0.86 (0.24) | 0.922 | 5.39 (26.23) | 0.63 (0.43) | **8.78E-03** | 537.65 (3157.2) | -26.50 (43.33) | **7.37E-03** |
| Busyness | 0.02 (0.01) | 0.02 (0.02) | 0.802 | 0.04 (0.14) | 0.03 (0.03) | 0.887 | 393.13 (2026.99) | 297.55 (621.41) | 0.196 |

Sd = standard deviation; SUV = standardized uptake values; TLG = total lesion glycolysis; CoV = coefficient of variation; MTV = metabolic tumor volume; GLCM = gray-level co-occurrence matrix; NGLDM = neighboring gray-level dependence matrix;

Bold values denote statistical significance at the *P* < 0.05 level.

^*^The percent change was calculated by 100 × [value at the first assessment (TP2) minus the baseline value (TP1)] / value at the baseline (TP1)

†*P* value was generated by *t*-test for parametric samples or Wilcoxon rank sum test for non-parametric samples, followed by correction for multiple comparisons by Benjamini-Hochberg method.

**Supplementary Table S3.** Univariate Cox regression analysis of pathoclinical variables for PFS and OS

| Clinical variables | **PFS** | | | **OS** | | |
| --- | --- | --- | --- | --- | --- | --- |
|  | HR | 95% CI | *P*^*^ | HR | 95% CI | *P*^*^ |
| Age (≥60 vs. <60 (ref)) | 0.7 | 0.45-1.09 | 0.113 | 0.66 | 0.41-1.05 | 0.081 |
| Gender (male vs. female (ref)) | 0.78 | 0.49-1.24 | 0.299 | 0.91 | 0.56-1.48 | 0.711 |
| HER2 status (positive vs. negative (ref)) | 0.88 | 0.49-1.56 | 0.653 | 0.92 | 0.50-1.69 | 0.79 |
| Pathology (poorly cohesive carcinoma vs. other (ref)) | 1.63 | 0.93-2.86 | 0.088 | 2.27 | 1.27-4.04 | **0.005** |
| BMI (≥25 vs. 20-24.9 (ref)) | 0.65 | 0.34-1.25 | 0.199 | 0.48 | 0.30-1.14 | 0.115 |
| BMI (<20 vs. 20-24.9 (ref)) | 1.02 | 0.62-1.69 | 0.924 | 0.8 | 0.46-1.38 | 0.419 |
| DM (yes vs. no (ref)) | 1.17 | 0.51-2.69 | 0.716 | 1.27 | 0.55-2.94 | 0.583 |
| Hypertension (yes vs. no (ref)) | 1.58 | 0.95-2.61 | 0.077 | 0.99 | 0.58-1.69 | 0.957 |
| ECOG performance status (1-2 vs. 0 (ref)) | 1.76 | 1.07-2.89 | **0.026** | 2.18 | 1.25-3.81 | **0.006** |
| CEA (≥10 vs. <10 (ref)) | 1.61 | 1.02-2.56 | **0.043** | 1.39 | 0.86-2.25 | 0.183 |
| CA 19-9 (≥24 vs. <24 (ref)) | 0.93 | 0.59-1.46 | 0.755 | 1.04 | 0.65-1.67 | 0.87 |
| Albumin (<3.3 vs. ≥3.3 (ref)) | 0.81 | 0.48-1.37 | 0.437 | 0.74 | 0.43-1.26 | 0.27 |
| Best response (non-responder vs. responder (ref)) | 1.96 | 1.24-3.09 | **0.004** | 3.31 | 2.01-5.46 | **2.84E-06** |
| Best response at TP2  (non-responder vs. responder (ref)) | 1.62 | 1.04-2.54 | **0.033** | 2.37 | 1.46-3.84 | **4.85E-04** |

PFS = progression-free survival; OS = overall survival; HER2 = human epidermal growth factor receptor 2; BMI = Body-mass index; ECOG = The Eastern Cooperative Oncology Group; CEA = Carcinoembryonic antigen; CA 19-9 = Carbohydrate antigen 19-9;

Bold values denote statistical significance at the *P* < 0.05 level.

^*^ *P* value was generated by Cox proportional hazards regression analysis.

**Supplementary Table S4**. Parameter ROC curves

|  |  | **PFS** | | **OS** | |
| --- | --- | --- | --- | --- | --- |
|  |  | Cut-off value | AUC-ROC | Cut-off value | AUC-ROC |
| **TP1 (baseline)** | |  |  |  |  |
|  | **Conventional Indices** |  |  |  |  |
|  | SUV_max_ | 5.945 | 0.503 | 5.994 | 0.533 |
|  | SUV_peak_ | 4.762 | 0.535 | 5.133 | 0.549 |
|  | TLG | 164.756 | 0.58 | 450.97 | 0.61 |
|  | CoV | 0.219 | 0.49 | 0.232 | 0.548 |
|  | MTV | 47.385 | 0.605 | 31.689 | 0.624 |
|  | **Histogram Indices** |  |  |  |  |
|  | Skewness | 0.247 | 0.46 | 0.329 | 0.455 |
|  | Kurtosis | 2.419 | 0.534 | 2.634 | 0.518 |
|  | Entropy _(log2)_ | 4.689 | 0.546 | 3.157 | 0.565 |
|  | Energy | 0.028 | 0.431 | 0.03 | 0.412 |
|  | **Shape Indices** |  |  |  |  |
|  | Sphericity | 0.879 | 0.493 | 0.904 | 0.534 |
|  | Compacity | 2.246 | 0.603 | 2.246 | 0.626 |
|  | **GLCM** |  |  |  |  |
|  | Contrast | 0.244 | 0.524 | 0.245 | 0.514 |
|  | Correlation | 0 | 0.494 | 0 | 0.461 |
|  | Entropy _(log2)_ | 0.182 | 0.492 | 0.299 | 0.557 |
|  | Dissimilarity | 1.671 | 0.576 | 2.682 | 0.588 |
|  | **NGLDM** |  |  |  |  |
|  | Coarseness | 534.304 | 0.596 | 558.45 | 0.604 |
|  | Contrast | 0.936 | 0.605 | 0.934 | 0.584 |
|  | Busyness | 0.004 | 0.536 | 0.024 | 0.499 |
| **TP2 (first assessment)** | |  |  |  |  |
|  | **Conventional Indices** |  |  |  |  |
|  | SUV_max_ | 6.362 | 0.736 | 6.362 | 0.739 |
|  | SUV_peak_ | 5.078 | 0.766 | 5.078 | 0.798 |
|  | TLG | 37.988 | 0.82 | 26.112 | 0.809 |
|  | CoV | 0.159 | 0.771 | 0.194 | 0.72 |
|  | MTV | 11.574 | 0.818 | 9.23 | 0.788 |
|  | **Histogram Indices** |  |  |  |  |
|  | Skewness | 0.262 | 0.672 | 0.356 | 0.607 |
|  | Kurtosis | 3.349 | 0.556 | 2.146 | 0.57 |
|  | Entropy _(log2)_ | 3.214 | 0.755 | 3.214 | 0.776 |
|  | Energy | 0.23 | 0.243 | 0.296 | 0.23 |
|  | **Shape Indices** |  |  |  |  |
|  | Sphericity | 0 | 0.57 | 0.813 | 0.576 |
|  | Compacity | 1.172 | 0.804 | 1.174 | 0.77 |
|  | **GLCM** |  |  |  |  |
|  | Contrast | 0 | 0.602 | 0 | 0.542 |
|  | Correlation | 0 | 0.546 | 0 | 0.508 |
|  | Entropy _(log2)_ | 0.433 | 0.824 | 0.256 | 0.766 |
|  | Dissimilarity | 1.755 | 0.791 | 1.835 | 0.796 |
|  | **NGLDM** |  |  |  |  |
|  | Coarseness | 187.123 | 0.818 | 188.347 | 0.777 |
|  | Contrast | 0 | 0.66 | 0.859 | 0.704 |
|  | Busyness | 0 | 0.502 | 0 | 0.519 |
| **Percent change** | |  |  |  |  |
|  | **Conventional Indices** |  |  |  |  |
|  | SUV_max_ | -55.219 | 0.668 | -16.166 | 0.686 |
|  | SUV_peak_ | -48.178 | 0.7 | -43.766 | 0.77 |
|  | TLG | -84.545 | 0.703 | -84.545 | 0.701 |
|  | CoV | -35.454 | 0.724 | -28.224 | 0.673 |
|  | MTV | -83.333 | 0.698 | -82.759 | 0.664 |
|  | **Histogram Indices** |  |  |  |  |
|  | Skewness | -29.788 | 0.614 | -23.544 | 0.552 |
|  | Kurtosis | 28.716 | 0.553 | -0.443 | 0.542 |
|  | Entropy _(log2)_ | -28.045 | 0.672 | -22.008 | 0.715 |
|  | Energy | -11.713 | 0.386 | -11.713 | 0.356 |
|  | **Shape Indices** |  |  |  |  |
|  | Sphericity | -5.548 | 0.513 | -6.134 | 0.554 |
|  | Compacity | -47.84 | 0.709 | -22.237 | 0.701 |
|  | **GLCM** |  |  |  |  |
|  | Contrast | -4.122 | 0.663 | -4.039 | 0.568 |
|  | Correlation | -15.766 | 0.665 | -15.766 | 0.571 |
|  | Entropy _(log2)_ | -33.883 | 0.747 | -29.361 | 0.716 |
|  | Dissimilarity | -29.774 | 0.697 | -23.731 | 0.723 |
|  | **NGLDM** |  |  |  |  |
|  | Coarseness | -82.17 | 0.75 | -66.013 | 0.726 |
|  | Contrast | -100 | 0.551 | -9.724 | 0.612 |
|  | Busyness | -100 | 0.479 | -35.791 | 0.513 |

ROC = receiver operating characteristics; AUC-ROC = area under the ROC curve; PFS = progression-free survival; OS = overall survival; SUV = standardized uptake values; TLG = total lesion glycolysis; CoV = coefficient of variation; MTV = metabolic tumor volume; GLCM = gray-level co-occurrence matrix; NGLDM = neighboring gray-level dependence matrix;

**Supplementary Table S5**. Univariate Cox regression analysis of radiomics variables for PFS and OS

| **Variables** | | **PFS** | | | **OS** | | |
| --- | --- | --- | --- | --- | --- | --- | --- |
|  |  | HR | 95% CI | *P*^*^ | HR | 95% CI | *P*^*^ |
| **TP1 (baseline)** | |  |  |  |  |  |  |
|  | **Conventional Indices** |  |  |  |  |  |  |
|  | SUV_max_ | 1.39 | 0.69-2.80 | 0.35 | 1.6 | 0.76-3.33 | 0.215 |
|  | SUV_peak_ | 2.1 | 1.04-4.23 | **0.038** | 1.93 | 0.92-4.03 | 0.0815 |
|  | TLG | 1.21 | 0.78-1.90 | 0.394 | 1.13 | 0.66-1.93 | 0.658 |
|  | CoV | 1.27 | 0.76-2.12 | 0.356 | 1.09 | 0.66-1.77 | 0.744 |
|  | MTV | 1.38 | 0.85-2.23 | 0.192 | 1.23 | 0.77-1.97 | 0.385 |
|  | **Histogram Indices** |  |  |  |  |  |  |
|  | Skewness | 1.13 | 0.68-1.89 | 0.632 | 1.09 | 0.66-1.80 | 0.743 |
|  | Kurtosis | 1.54 | 0.88-2.68 | 0.128 | 1.2 | 0.73-1.96 | 0.47 |
|  | Entropy _(log2)_ | 0.94 | 0.58-1.53 | 0.807 | 1.43 | 0.65-3.12 | 0.371 |
|  | Energy | 1.83 | 0.74-4.55 | 0.192 | 1.33 | 0.61-2.91 | 0.478 |
|  | **Shape Indices** |  |  |  |  |  |  |
|  | Sphericity | 1.37 | 0.77-2.41 | 0.281 | 1.22 | 0.72-2.07 | 0.467 |
|  | Compacity | 1.34 | 0.82-2.19 | 0.238 | 1.55 | 0.94-2.55 | 0.087 |
|  | **GLCM** |  |  |  |  |  |  |
|  | Contrast | 2.29 | 1.21-4.34 | **0.011** | 2.1 | 1.15-3.81 | **0.015** |
|  | Correlation | *omitted^†^* |  |  | *omitted^†^* |  |  |
|  | Entropy _(log2)_ | 3.89 | 0.93-16.24 | 0.063 | 2.1 | 1.00-4.41 | **0.049** |
|  | Dissimilarity | 1.81 | 0.82-3.98 | 0.142 | 1.31 | 0.76-2.26 | 0.331 |
|  | **NGLDM** |  |  |  |  |  |  |
|  | Coarseness | 1.36 | 0.87-2.12 | 0.176 | 1.37 | 0.86-2.2 | 0.186 |
|  | Contrast | 1.62 | 1.04-2.54 | **0.035** | 1.45 | 0.91-2.32 | 0.118 |
|  | Busyness | 1.72 | 0.91-3.23 | 0.0945 | 1.5 | 0.89-2.53 | 0.126 |
| **TP2 (first assessment)** | |  |  |  |  |  |  |
|  | **Conventional Indices** |  |  |  |  |  |  |
|  | SUV_max_ | 3.09 | 1.78-5.37 | **6.37E-05** | 3.27 | 1.80-5.97 | **1.10E-04** |
|  | SUV_peak_ | 3.32 | 1.92-5.74 | **1.83E-05** | 4.27 | 2.34-7.77 | **2.07E-06** |
|  | TLG | 3.33 | 1.89-5.85 | **3.01E-05** | 3.52 | 1.81-6.85 | **2.19E-04** |
|  | CoV | 3.21 | 1.65-6.25 | **5.95E-04** | 2.54 | 1.48-4.35 | **7.36E-04** |
|  | MTV | 3.2 | 1.83-5.60 | **4.38E-05** | 3.55 | 1.99-6.31 | **1.73E-05** |
|  | **Histogram Indices** |  |  |  |  |  |  |
|  | Skewness | 1.1 | 0.67-1.78 | 0.712 | 1.58 | 0.95-2.64 | 0.083 |
|  | Kurtosis | 1.23 | 0.68-2.22 | 0.492 | 1.79 | 0.25-12.98 | 0.563 |
|  | Entropy _(log2)_ | 3.31 | 1.95-5.62 | **9.23E-06** | 3.37 | 1.92-5.92 | **2.48E-05** |
|  | Energy | 0.87 | 0.21-3.57 | 0.845 | 1.63 | 0.22-11.93 | 0.631 |
|  | **Shape Indices** |  |  |  |  |  |  |
|  | Sphericity | *omitted^†^* |  |  | 2.47 | 1.27-4.80 | **7.54E-03** |
|  | Compacity | 2.68 | 1.55-4.61 | **3.91E-04** | 2.73 | 1.57-4.75 | **3.77E-04** |
|  | **GLCM** |  |  |  |  |  |  |
|  | Contrast | *omitted^†^* |  |  | *omitted^†^* |  |  |
|  | Correlation | *omitted^†^* |  |  | *omitted^†^* |  |  |
|  | Entropy _(log2)_ | 3.01 | 1.75-5.17 | **6.95E-05** | 2.83 | 1.51-5.29 | **1.17E-03** |
|  | Dissimilarity | 4.2 | 2.36-7.47 | **1.02E-06** | 4.35 | 2.49-7.60 | **2.46E-07** |
|  | **NGLDM** |  |  |  |  |  |  |
|  | Coarseness | 3.88 | 2.19-6.86 | **3.29E-06** | 4.35 | 2.45-7.74 | **5.67E-07** |
|  | Contrast | *omitted^†^* |  |  | 2.51 | 1.26-4.99 | **8.87E-03** |
|  | Busyness | *omitted^†^* |  |  | *omitted^†^* |  |  |
| **Percent change** | |  |  |  |  |  |  |
|  | **Conventional Indices** |  |  |  |  |  |  |
|  | SUV_max_ | 2.71 | 1.47-5.01 | **0.001** | 2.84 | 1.62-4.99 | **0.000272** |
|  | SUV_peak_ | 2.46 | 1.38-4.40 | **0.002** | 3.79 | 2.11-6.83 | **8.84E-06** |
|  | TLG | 2.18 | 1.24-3.86 | **0.007** | 3.28 | 1.72-6.26 | **3.11E-04** |
|  | CoV | 3.36 | 1.88-6.02 | **4.60E-05** | 3.11 | 1.74-5.58 | **1.36E-04** |
|  | MTV | 2.85 | 1.47-5.53 | **0.002** | 3.92 | 1.83-8.39 | **4.49E-04** |
|  | **Histogram Indices** |  |  |  |  |  |  |
|  | Skewness | 1.24 | 0.77-2.02 | 0.379 | 1.88 | 1.12-3.14 | **0.016** |
|  | Kurtosis | 1.66 | 0.87-3.16 | 0.124 | 1.47 | 0.87-2.46 | 0.146 |
|  | Entropy _(log2)_ | 2.96 | 1.68-5.22 | **1.70E-04** | 3.36 | 1.91-5.89 | **2.38E-05** |
|  | Energy | 1.14 | 0.54-2.39 | 0.733 | 1.45 | 0.62-3.37 | 0.39 |
|  | **Shape Indices** |  |  |  |  |  |  |
|  | Sphericity | 1.83 | 1.05-3.20 | **0.035** | 2.26 | 1.13-4.50 | **0.021** |
|  | Compacity | 2.29 | 1.27-4.11 | **5.61E-03** | 2.72 | 1.59-4.64 | **2.44E-04** |
|  | **GLCM** |  |  |  |  |  |  |
|  | Contrast | 2.17 | 1.23-3.83 | **7.28E-03** | 2 | 1.10-3.62 | **0.023** |
|  | Correlation | 2.18 | 1.23-3.88 | **8.07E-03** | 2.44 | 1.29-4.65 | **6.39E-03** |
|  | Entropy _(log2)_ | 1.96 | 1.18-3.24 | **9.38E-03** | 2.29 | 1.33-3.95 | **0.003** |
|  | Dissimilarity | 2.76 | 1.48-5.14 | **1.43E-03** | 3.91 | 2.18-7.00 | **4.59E-06** |
|  | **NGLDM** |  |  |  |  |  |  |
|  | Coarseness | 3.05 | 1.69-5.51 | **2.22E-04** | 3.43 | 1.93-6.10 | **2.72E-05** |
|  | Contrast | *omitted^†^* |  |  | 2.61 | 1.17-5.81 | **0.019** |
|  | Busyness | *omitted^†^* |  |  | 1.62 | 0.85-3.08 | 0.139 |

PFS = progression-free survival; OS = overall survival; HR = hazard ratio; CI = confidence interval; HER2 = Human epidermal growth factor receptor 2; BMI = body-mass index; DM = diabetes mellitus; ECOG = Eastern Cooperative Oncology Group; CEA = Carcinoembryonic antigen; CA 19-9 = carbohydrate antigen 19-9; SUV = standardized uptake values; TLG = total lesion glycolysis; CoV = coefficient of variation; MTV = metabolic tumor volume; GLCM = gray-level co-occurrence matrix; NGLDM = neighboring gray-level dependence matrix;

Bold values denote statistical significance at the *P* < 0.05 level.

^*^ *P* value was generated by Cox proportional hazards regression analysis.

^†^The variable was omitted from the univariate Cox regression analysis because of perfect prediction.

**Supplementary Table S6.** Performance of models

|  | **PFS** | | **OS** | |
| --- | --- | --- | --- | --- |
| Model | C-index | 95% CI^*^ | C-index | 95% CI^*^ |
| Pathoclinical | 0.686 | 0.573-0.776 | 0.74 | 0.693-0.802 |
| Radiomics |  |  |  |  |
| TP1 | 0.635 | 0.500-0.730 | 0.671 | 0.549-0.767 |
| TP2 | 0.717 | 0.644-0.784 | 0.701 | 0.646-0.767 |
| delta | 0.695 | 0.645-0.787 | 0.747 | 0.694-0.819 |
| TP2 + delta | 0.755 | 0.702-0.844 | 0.767 | 0.709-0.849 |
| Pathoclinical + Radiomics (TP2 + delta) | 0.772 | 0.717-0.862 | 0.799 | 0.762-0.890 |

PFS = progression-free survival; OS = overall survival; C-index = concordance index; CI = confidence interval;

^*^Calculated from bootstrapping (500 iterations)

**Supplementary Table S7. Performance of model in 77 patients who did not progress at TP2.**

|  | **PFS** | | **OS** | |
| --- | --- | --- | --- | --- |
| Model | C-index | 95% CI^*^ | C-index | 95% CI^*^ |
| Pathoclinical | 0.681 | 0.500-0.777 | 0.721 | 0.688-0.796 |
| Radiomics |  |  |  |  |
| TP1 | 0.619 | 0.500-0.725 | 0.657 | 0.500-0.758 |
| TP2 | 0.722 | 0.640-0.804 | 0.699 | 0.634-0.773 |
| Delta | 0.691 | 0.605-0.793 | 0.737 | 0.674-0.833 |
| TP2 + delta | 0.758 | 0.678-0.852 | 0.758 | 0.705-0.857 |
| Pathoclinical + Radiomics (TP2 + delta) | 0.775 | 0.711-0.871 | 0.814 | 0.754-0.908 |

PFS = progression-free survival; OS = overall survival; C-index = concordance index; CI = confidence interval;

^*^Calculated from bootstrapping (500 iterations)

**Supplementary Table S8**. Texture analysis parameter types

| **Category** | **Parameter** | **Description** |
| --- | --- | --- |
| **Histogram Indices** | Skewness | measures the asymmetry of the grey-level distribution in the histogram |
|  | Kurtosis | measures whether the grey-level distribution is peaked of flat relative to a normal distribution |
|  | Entropy | measures the randomness of the distribution |
|  | Energy | measures the uniformity of the distribution |
| **Shape Indices** | Sphericity | measures how spherical a volume of interest is |
|  | Compacity | measures the degree to which the volume of interest is compact |
| **GLCM** | Correlation | linear dependency of grey-levels in GLCM |
|  | Contrast | local variations in the GLCM |
|  | Entropy | randomness of grey-level voxel pairs |
|  | Dissimilarity | variation of grey-level voxel pairs |
| **NGLDM** | Coarseness | level of spatial rate of change in intensity |
|  | Contrast | intensity difference between neighboring regions |
|  | Busyness | spatial frequency of changes in intensity |

GLCM = gray-level co-occurrence matrix; NGLDM = neighboring gray-level dependence matrix;

**Supplementary Table S9**. TRIPOD Checklist: Prediction model development

| Section/Topic | Item | Checklist Item | Page |
| --- | --- | --- | --- |
| Title and abstract | | | |
| Title | 1 | Identify the study as developing and/or validating a multivariable prediction model, the target population, and the outcome to be predicted. | 1 |
| Abstract | 2 | Provide a summary of objectives, study design, setting, participants, sample size, predictors, outcome, statistical analysis, results, and conclusions. | 2 |
| Introduction | | | |
| Background and objectives | 3a | Explain the medical context (including whether diagnostic or prognostic) and rationale for developing or validating the multivariable prediction model, including references to existing models. | 3 |
|  | 3b | Specify the objectives, including whether the study describes the development or validation of the model or both. | 4 |
| Methods | | | |
| Source of data | 4a | Describe the study design or source of data (e.g., randomized trial, cohort, or registry data), separately for the development and validation data sets, if applicable. | 10 |
|  | 4b | Specify the key study dates, including start of accrual; end of accrual; and, if applicable, end of follow-up. | 10 |
| Participants | 5a | Specify key elements of the study setting (e.g., primary care, secondary care, general population) including number and location of centres. | 10 |
|  | 5b | Describe eligibility criteria for participants. | 10 |
|  | 5c | Give details of treatments received, if relevant. | 10 |
| Outcome | 6a | Clearly define the outcome that is predicted by the prediction model, including how and when assessed. | 11-12 |
|  | 6b | Report any actions to blind assessment of the outcome to be predicted. | 11 |
| Predictors | 7a | Clearly define all predictors used in developing or validating the multivariable prediction model, including how and when they were measured. | 11-12 |
|  | 7b | Report any actions to blind assessment of predictors for the outcome and other predictors. | 10 |
| Sample size | 8 | Explain how the study size was arrived at. | NA |
| Missing data | 9 | Describe how missing data were handled (e.g., complete-case analysis, single imputation, multiple imputation) with details of any imputation method. | 12-13 |
| Statistical analysis methods | 10a | Describe how predictors were handled in the analyses. | 12-13 |
|  | 10b | Specify type of model, all model-building procedures (including any predictor selection), and method for internal validation. | 12-13 |
|  | 10d | Specify all measures used to assess model performance and, if relevant, to compare multiple models. | 12-13 |
| Risk groups | 11 | Provide details on how risk groups were created, if done. | 12-13 |
| Results | | | |
| Participants | 13a | Describe the flow of participants through the study, including the number of participants with and without the outcome and, if applicable, a summary of the follow-up time. A diagram may be helpful. | 4 |
|  | 13b | Describe the characteristics of the participants (basic demographics, clinical features, available predictors), including the number of participants with missing data for predictors and outcome. | 4, 20 |
| Model development | 14a | Specify the number of participants and outcome events in each analysis. | 4 |
|  | 14b | If done, report the unadjusted association between each candidate predictor and outcome. | 5 |
| Model specification | 15a | Present the full prediction model to allow predictions for individuals (i.e., all regression coefficients, and model intercept or baseline survival at a given time point). | 5-6 |
|  | 15b | Explain how to the use the prediction model. | 5-6 |
| Model performance | 16 | Report performance measures (with CIs) for the prediction model. | 5-6 |
| Discussion | | | |
| Limitations | 18 | Discuss any limitations of the study (such as nonrepresentative sample, few events per predictor, missing data). | 9 |
| Interpretation | 19b | Give an overall interpretation of the results, considering objectives, limitations, and results from similar studies, and other relevant evidence. | 6-7 |
| Implications | 20 | Discuss the potential clinical use of the model and implications for future research. | 8-9 |
| Other information | | | |
| Supplementary information | 21 | Provide information about the availability of supplementary resources, such as study protocol, Web calculator, and data sets. | 4-6 |
| Funding | 22 | Give the source of funding and the role of the funders for the present study. | 13 |
