# Supplementary figures and images for: Prospective evaluation of metabolic intratumoral heterogeneity in patients with advanced gastric cancer receiving palliative chemotherapy
Source: Sci Rep. 2021 Jan 12;11:296. doi: 10.1038/s41598-020-78963-2 (PMC7804009; doi:10.1038/s41598-020-78963-2)

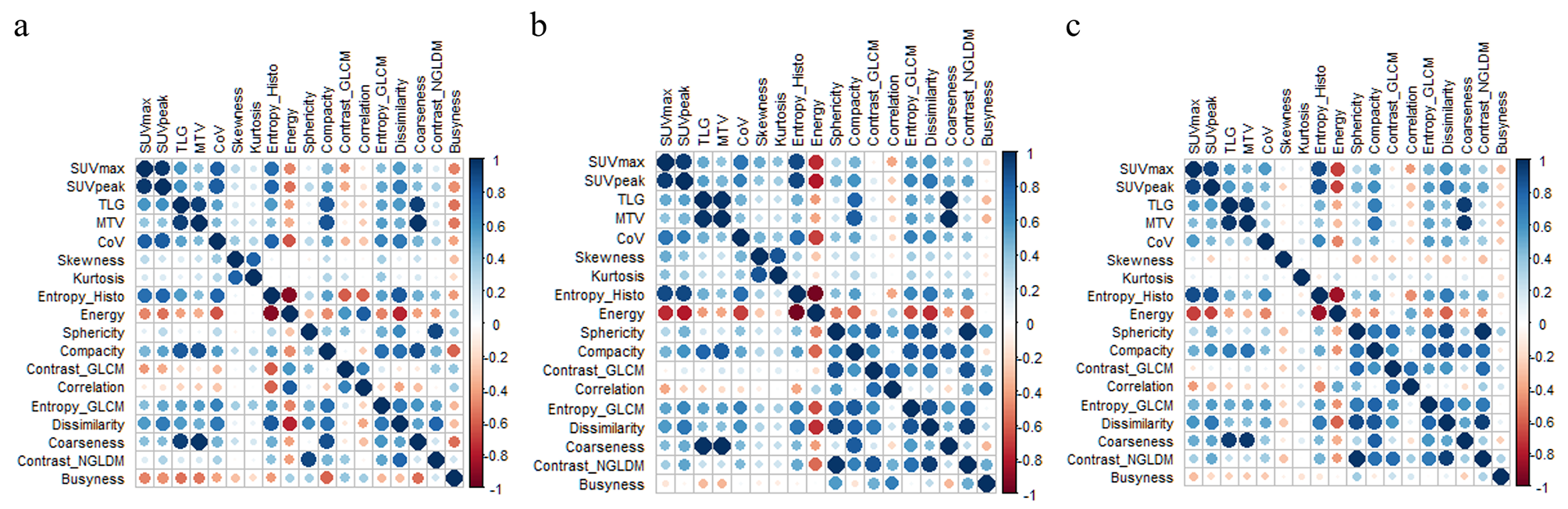

Supplement: Supplementary file 2 — Supplementary Figure S1. [file 41598_2020_78963_MOESM2_ESM.tif]

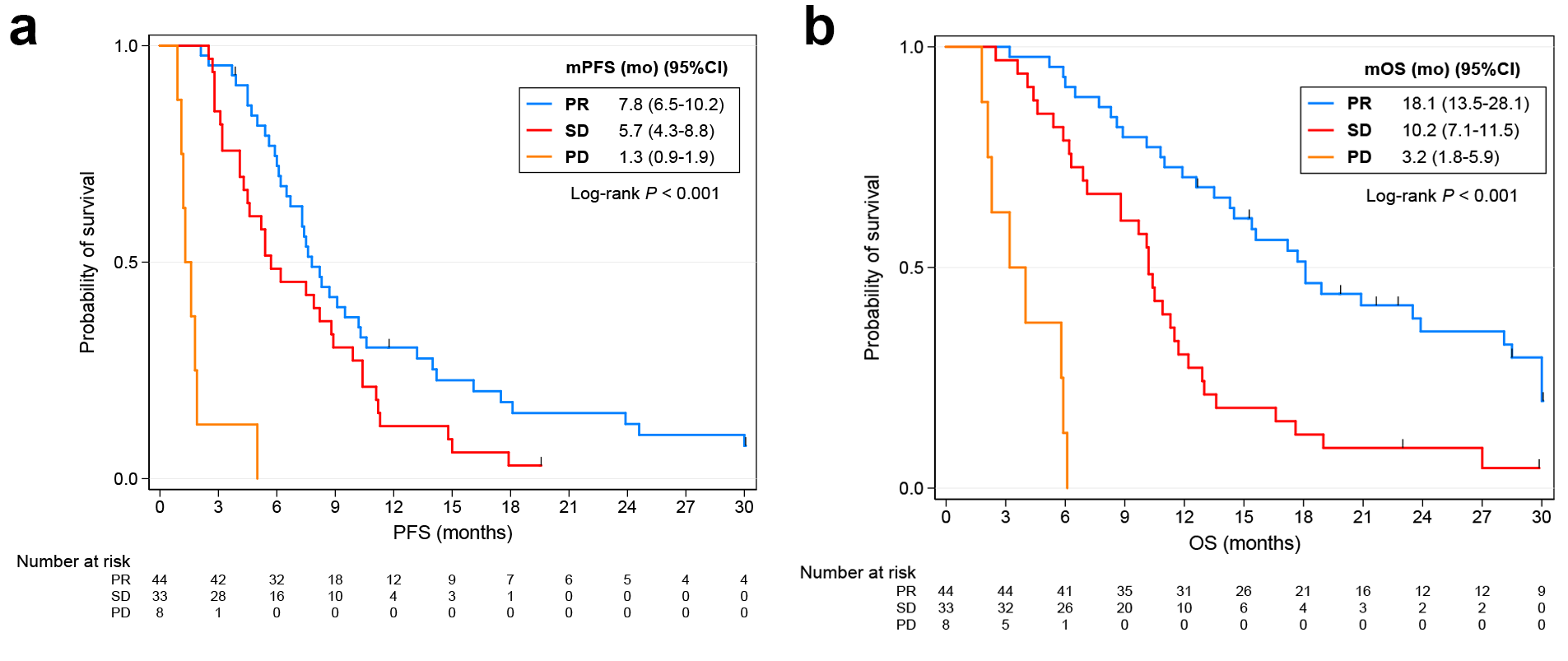

Supplement: Supplementary file 3 — Supplementary Figure S2. [file 41598_2020_78963_MOESM3_ESM.tif]

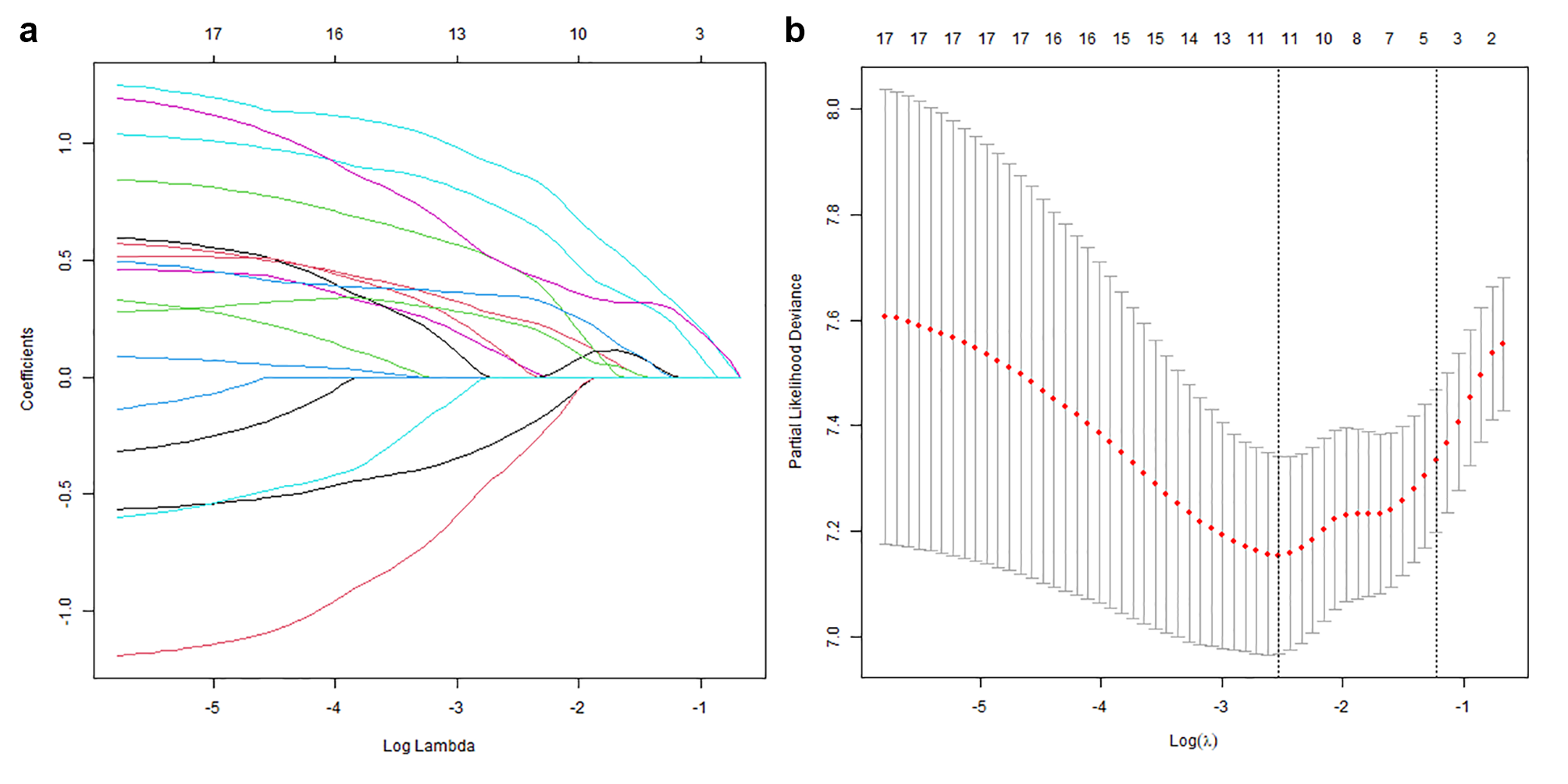

Supplement: Supplementary file 4 — Supplementary Figure S3. [file 41598_2020_78963_MOESM4_ESM.tif]

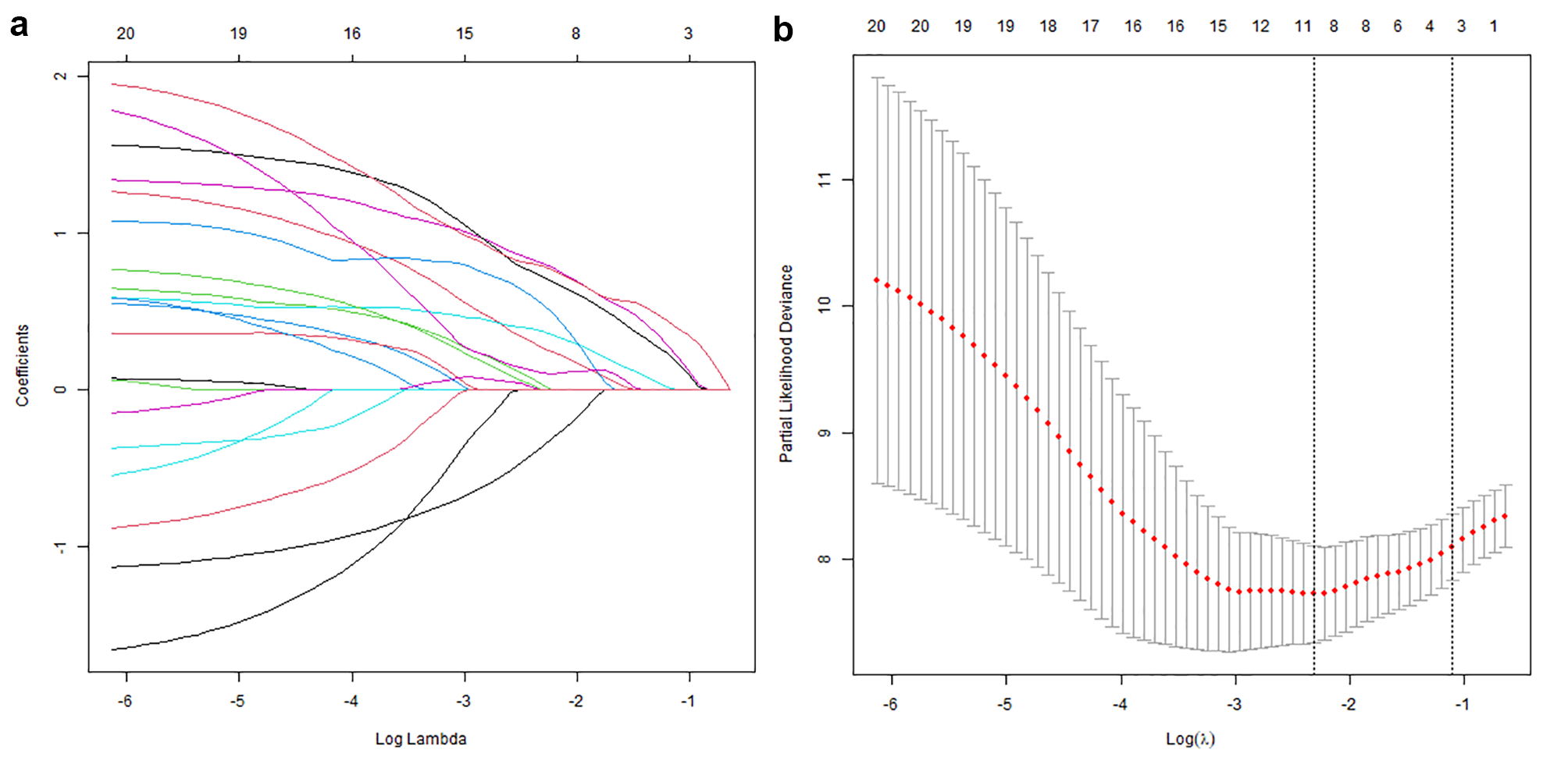

Supplement: Supplementary file 5 — Supplementary Figure S4. [file 41598_2020_78963_MOESM5_ESM.tif]

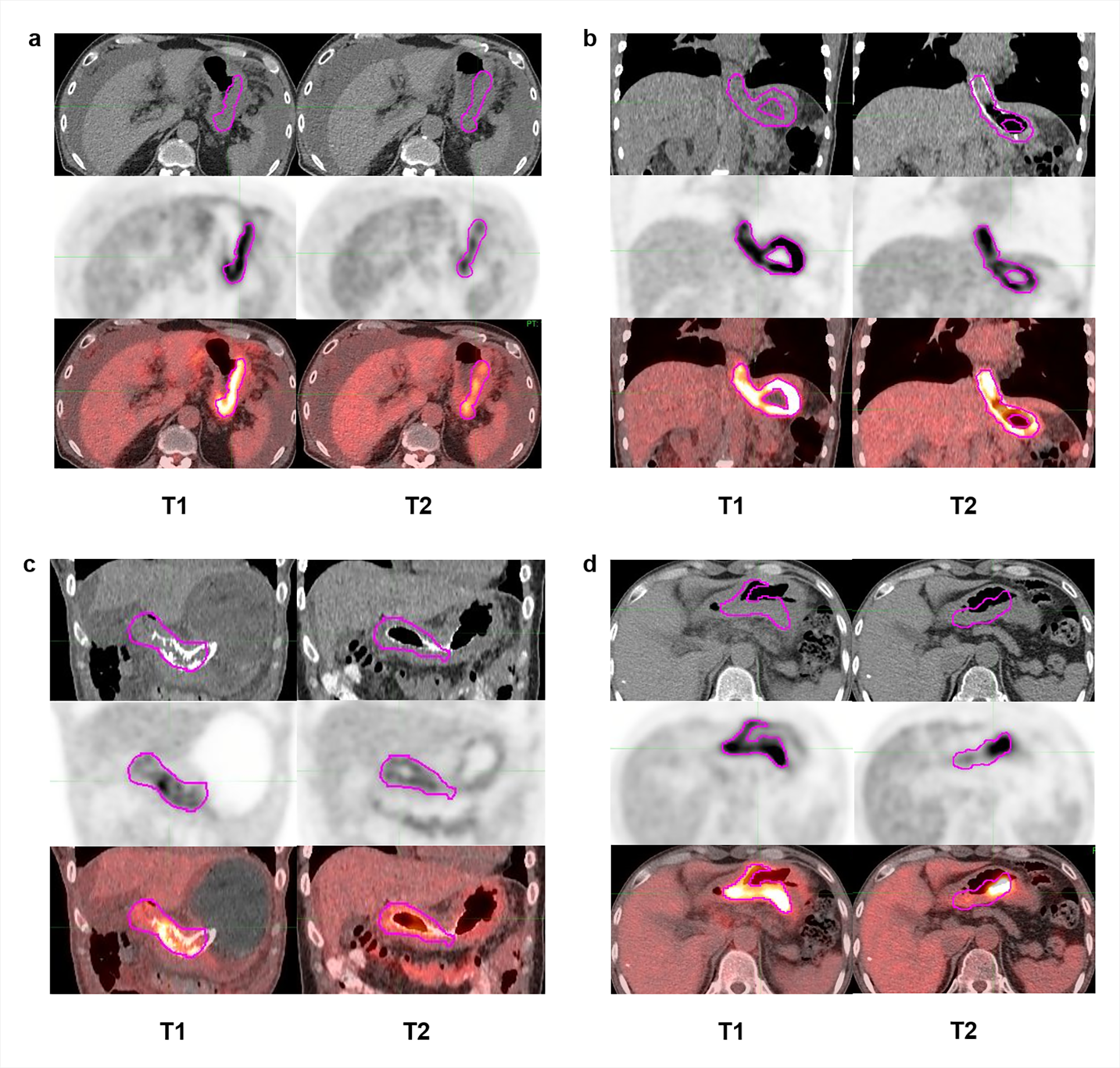

Supplement: Supplementary file 6 — Supplementary Figure S5. [file 41598_2020_78963_MOESM6_ESM.tif]
